# Clinical Perspectives and Fundamental Aspects of Local Cardiovascular and Renal Renin-Angiotensin Systems

**DOI:** 10.3389/fendo.2014.00016

**Published:** 2014-02-19

**Authors:** Walmor C. De Mello, Edward D. Frohlich

**Affiliations:** ^1^School of Medicine, University of Puerto Rico Medical Sciences Campus, San Juan, PR, USA; ^2^Alton Ochsner Medical Foundation, New Orleans, LA, USA

**Keywords:** local renin-angiotensin systems, heart, arteries and kidney

## Abstract

Evidence for the potential role of organ specific cardiovascular renin–angiotensin systems (RAS) has been demonstrated experimentally and clinically with respect to certain cardiovascular and renal diseases. These findings have been supported by studies involving pharmacological inhibition during ischemic heart disease, myocardial infarction, cardiac failure; hypertension associated with left ventricular ischemia, myocardial fibrosis and left ventricular hypertrophy; structural and functional changes of the target organs associated with prolonged dietary salt excess; and intrarenal vascular disease associated with end-stage renal disease. Moreover, the severe structural and functional changes induced by these pathological conditions can be prevented and reversed by agents producing RAS inhibition (even when not necessarily coincident with alterations in arterial pressure). In this review, we discuss specific fundamental and clinical aspects and mechanisms related to the activation or inhibition of local RAS and their implications for cardiovascular and renal diseases. Fundamental aspects involving the role of angiotensins on cardiac and renal functions including the expression of RAS components in the heart and kidney and the controversial role of angiotensin-converting enzyme 2 on angiotensin peptide metabolism in humans, were discussed.

## Introduction

The presence of local organ specific renin–angiotensin systems (RAS) has been demonstrated for the heart, large arteries and arterioles, kidneys, and other organs and their activation lead to structural and functional changes, which are independent of those elicited by the classical renin–angiotensin endocrine system (1–4). Components of these local RAS, for instance, have been found in cells and tissues (5–8) and some of their local functions play an important role on cellular homeostasis.

In this review, we present several clinical circumstances involving certain cardiovascular diseases, which support the notion that the activation of local RAS plays an important role on the mechanisms of these pathological conditions. These vignettes cited also involve renal diseases because the renal glomerular and arteriolar alterations contribute to the development and progression of end-stage renal disease (ESRD).

## Clinical Circumstances

### Myocardial infarction and cardiac failure

This first clinical cardiovascular local RAS example relates to the introduction of angiotensin-converting enzyme (ACE) inhibitors and later to angiotensin II (type 1) receptor blocking agents (ARBs) to patients hospitalized with an initial myocardial infarction. This innovative therapeutic intervention proved to reduce ventricular remodeling in naturally developing spontaneously hypertensive rats (SHRs) (9) and following myocardial infarction in rats (10) then later in a small number of hospitalized patients (11) and, ultimately, in a larger clinical trial involving patients enrolled in the survival and ventricular enlargement (SAVE) trial (12). Thus, in patients who were promptly treated with an ACE inhibitor, immediately following acute myocardial infarction, a significant reduction in death, development of heart failure, and subsequent repeated myocardial infarction were found. Several subsequent multicenter clinical trials, using other ACE inhibitors or the newer ARBs, confirmed the initial findings thereby demonstrating their beneficial effects on ventricular remodeling, reduction in the end-stage events of cardiac failure, and repeated myocardial infarction (13). The finding that these beneficial effects can occur independently of blood pressure supports the conclusion that the activation of local RAS contributes significantly to cardiovascular pathology (14).

### Hypertensive heart disease

Similar evidence involving therapeutic intervention was demonstrated by the findings of the initial Veterans Administration Cooperative Study Treatment Group on Antihypertensive Agents (15, 16) and by the Framingham Heart Study’s first demonstration of “Factors of Risk” underlying coronary heart disease (17). The existence that cardiac failure and left ventricular hypertrophy (LVH), respectively, were first introduced by these two groups, to interdict in the major cardiac fatal and treatable complications of hypertensive heart disease (15–17). Subsequent reports later demonstrated that these two major complications of hypertensive heart disease were prevented by antihypertensive therapy. They also introduced the means to reduce left ventricular (LV) mass and its co-morbid events (18). In more recent years, increased LV mass and LVH were shown to be associated with extensive interstitial and perivascular fibrosis as well as by significant ischemia of both ventricles (18–20). Furthermore, when patients with LVH associated with hypertension (but not by co-existent occlusive coronary artery disease) were also treated with RAS inhibitors, the fibrosis and ischemia were significantly reduced (18–22). This, then, provided additional evidence of the beneficial value of local cardiac RAS inhibition.

The precise mechanisms underlying the development of LVH have usually been explained as an adaptive compensation by the LV to pressure overload by the hypertensive disease. Newer information has been introduced more recently concerning the development of fibrosis, apoptosis, aldosterone, and other induced cellular biochemical events in the LV. Others have suggested that angiotensin II (Ang II) causes hypertension and LVH through actions of AT1 receptors expressed by the kidney that reduce urinary sodium excretion (23) not involving Ang II-mediated aldosterone responses.

### Prolonged dietary salt excess

Two very different diseases involving local RAS in the heart as well as in the glomerular arterioles of the kidneys (24–26) support the important role of local RAS. SHRs receiving long-term dietary salt excess have shown remarkably similar pathophysiological expressions of disease similar to those which occurs in patients with hypertension having ventricular fibrosis, myocardial ischemia, and heart failure or with ESRD (27–29). These end-stage events occur in patients with hypertension and/or with diabetes mellitus having ESRD with intrarenal fibrosis and hyaline degeneration of the glomerulae and arterioles. As with the foregoing diseases that were shown by controlled multicenter drug trials using Ang II inhibitors described above, the progression of ESRD was also shown to be significantly retarded (30, 31). Interestingly, a recent report of SHR, given a prolonged dietary excess of salt, demonstrated a second local renal RAS (in addition to that of the juxtaglomerular apparatus) that produced a more plentiful production of angiotensinogen (32).

Finally, a word of speculation may be in order involving end-stage cardiac and renal diseases, the most common causes of hospitalization in geriatric hypertensive (or normotensive) patients from industrialized nations (33). These data suggest that a lifetime of excessively high salt intake together with these untoward outcomes may be intimately associated with the aging process (34). Indeed perhaps this may also relate to our new knowledge about local RAS in heart, arteries and arterioles of the kidneys.

## Fundamental Aspects Behind the Foregoing Clinical Examples

Experimental evidence supporting the notion that local RAS are present in different organs including the heart and kidney (3, 4, 35–40) has opened a new window into our understanding of how the local RASs contributes to local regulation of tissue and organ function. The synthesis of several components of the RAS in the heart (8, 41) or their uptake from plasma (8, 36, 41), for instance, makes it possible to explain the synthesis of Ang II locally (41). Furthermore, the presence of AT1 receptors, angiotensinogen and Ang II in different cells (8), supports the concept of local RAS. In the normal heart of pigs as much as 75% of cardiac Ang II is synthesized at tissue sites (42) whereas in human beings, the gradients of Ang II across the heart were increased in patients with congestive heart failure (5). Rapid internalization of the Ang II–AT1 receptor complex, contributes significantly to the intracellular levels of the peptide [see for review Ref. (43)] and the internalized AT1 receptor, is displaced to different organelles including the nucleus and mitochondria (43–47). Activation of AT1 receptor binding sites in renal nuclei has been found to elicit an increase in calcium (48) and in the expression of TGF-B1 and NHE-3 (46). Concerning the role of the local renal RAS on the generation of hypertension, recent studies revealed that the infusion of Ang II into mice lacking renal ACE, indicated no renal responses or hypertension in the knockout mice compared with wild-type control (49).

Transgenic mouse models developed to examine the role of the local RAS on cardiac remodeling, generated contradictory results revealing ventricular hypertrophy or fibrosis in some models but not in other (40, 50, 51) leading to the conclusion that cardiac remodeling is probably much more dependent on hemodynamic changes than on local Ang II levels. In hypertensive transgenic mouse lacking the synthesis of angiotensinogen, for instance, the local components of the RAS do not seem to be essential for the subsequent development of ventricular hypertrophy and fibrosis (41). The production of Ang II, in cardiac muscle caused by a αMHC promoter, increases the release of Ang II by 20-fold, but not hypertrophy was produced (51). On the other hand, in transgenic mouse lines over-expressing angiotensinogen by the heart, Ang II is increased in cardiac muscle but not in plasma (52) and ventricular hypertrophy was found despite no change in blood pressure. In these models, the hypertrophy was abolished by ACE inhibitors or AT1 blockers (53), again supporting the notion of a local RAS. Xu et al. (54) found that when hemodynamic loading conditions remain unchanged, cardiac Ang II does not elicit hypertrophy but in animals with hypertension, cardiac Ang II, acting via AT(1)R, increases oxidative stress, inflammation, ventricular hypertrophy, and cell death (probably via down regulation of PI 3 kinase and Akt).

These apparent discrepant results achieved with different transgenic models could be related to the use of different animal species or experimental conditions. Furthermore, the only parameter used to define cardiac remodeling in many of these studies was ventricular hypertrophy and other aspects of cellular remodeling like cell communication, fibrosis as well as expression and function of ionic channels were not considered.

Concerning the origin of cardiac renin, evidence is available that in the normal heart, cardiac renin is dependent on its uptake from plasma (6, 36, 42) but studies performed after myocardial infarction (55) or after stretch of cardiomyocytes (40) showed increased renin expression. Furthermore, a renin transcript that does not encode a secretory signal and remains inside the cell is over-expressed during myocardial infarction (55, 56) suggesting that intracellular renin has functional properties. The cytosolic renin protein exerts functions different and even opposite to those of secretory renin, which increases necrotic death rates of cardiac cells, while the cytosolic renin isoform even protects cells from necrotic death (56). In adrenal gland, a local secretory RAS exists that may stimulate aldosterone production and elicits an amplification for circulating angiotensin (Ang II) (57). The regulation of the secretory adrenal RAS is clearly different from the regulation of the circulatory RAS because under potassium load, the activity of the renal and circulatory RAS is suppressed whereas activity of the adrenal RAS is stimulated (57).

The function of intracellular renin and Ang II was demonstrated when renin or Ang II was dialyzed into cardiac myocytes from the failing heart. Renin, and particularly Ang II, decreased cell communication and increased the inward calcium current (37, 58, 59). The decrease of gap junction conductance leads to a decrease of electrical coupling and mechanical desynchronization as well as the generation of slow conduction and cardiac arrhythmias (60, 61). Recent studies performed on the intact ventricle of normal rats revealed that intracellular renin causes a depolarization of ventricular fibers and a decreased action potential duration at 50 and 90% repolarization, respectively while the cardiac refractoriness was significantly decreased with consequent generation of triggered activity (59). The intimate mechanism by which intracellular renin alters cardiac excitability involves changes of potassium current, which is responsible for repolarization of the action potential (59). The possible role of an intracellular renin receptor (62), which is activated by renin (62, 63), cannot be discarded and further studies will be needed to support this idea. The pathophysiological significance of intracellular renin is far from clear and further studies will be needed to clarify this point.

## Recent Developments

Our view of the RAS has been changed dramatically in recent years with studies demonstrating that Ang II can be hydrolyzed by angiotensin-converting enzyme 2 (ACE2), angiotensinases as well as neprilysin generating angiotensin (1–7) [Ang (1–7)], Ang A, Ang IV, and Ang III (64–66) and that new receptors for Ang (IV) (AT4), prorenin [(pro)renin receptor (PRR)], and Mas receptor for Ang (1–7) have been identified (67–70). Interestingly, the activation of prorenin receptor is able not only to catalyze prorenin to Ang II but also to induce cellular responses not related to the peptide (71, 72). Of particular interest is the recent finding that not all the peptides from RAS are derived from Ang I. The plasma levels of Ang (1–12), initially isolated from the rat intestine and present in heart, aorta, and kidney (73, 74) are not altered by renin inhibition or bilateral nephrectomy, which suggests a local effect of Ang (1–12) in tissues independently of the systemic circulation (73, 74). Chymase seems to be the most important enzyme involved in the metabolism of Ang (1–12), at least in the heart (75). Other studies of Ang (1–12) metabolism indicated that in the plasma of normal or hypertensive rats, ACE has a role generating Ang I from Ang (1–12) (76).

A new component of the RAS is amantadine, which is a heptapeptide possessing functions similar to those of Ang (1–7) and found in human plasma particularly in patients with ESRDs (77). The vasodilation caused by amantadine was not inhibited in Mas-deficient mice (77) suggesting its interaction with another Mas receptor. The precise role of this compound on cardiovascular disease is not known.

### AT2 receptors

Although it is known that the effect of Ang II on cardiac and vascular remodeling involves the activation of AT1 receptors, recent studies revealed that the AT2 receptor activation causes vasodilation and its agonist C21 is able to decrease myocardial fibrosis and vascular injury in SHRs [see Ref. (66, 78)]. The role of AT2 receptors on cardiac remodeling is supported by studies using AT2-knockout mice and the results indicated that this receptor plays an essential role in the development of ventricular hypertrophy induced by pressure overload (79) [see Ref. (80)]. AT2 receptor activation seems to inhibit inflammation and apoptosis (81), attenuates cardiopulmonary injury by decreasing pulmonary inflammation (82) and in obese animals, long-term activation of AT2 receptors increases ACE2 activity and contributes to natriuresis and blood pressure reduction (83). The natriuresis is probably related to Ang III (84).

Myocardial fibrosis impairs ventricular relaxation and is an important cause of diastolic heart failure. The presence of fibrosis is not limited to the left ventricle and is found in the right ventricle as well as in the interventricular septum, suggesting that hypertension is not the only factor involved but also local production of Ang II is involved (85). The fibrotic action of the peptide within the heart seems to depend on fibroblast hyperplasia as well as activation of collagen biosynthesis and suppression of collagen degradative pathways. Activation of pathways related to AT1 receptors as well as MAP/endoplasmic reticulum (ER) kinase pathway activation play a key role of the generation of fibrosis and recently, evidence has been provided that Ang II AT2 receptors prevent cardiac remodeling after myocardial infarction and improve cardiac function (86).

### The (pro)renin receptor

The PRR (71), mainly located intracellularly (62), is a new member of the RAS, originally considered as involved in the regulation of blood pressure. Recent observations using transgenic animals over-expressing PRR did not provide a clear answer to this question but demonstrated different aspects of PRR biology. It is now clear that PRR is an accessory protein of V-ATPase (87) playing an important role on the regulation of several cellular homeostatic processes including autophagy (88).

A knockout model generated by Kinouchi et al. (89) showed death within 3 weeks and an accumulation of vesicles and autophagosomes in cardiomyocytes indicating a change in autophagic flux. The role of PRR on the etiology of cardiovascular diseases, however, is not clear and further studies will be needed to clarify this point.

### Adipocytes and regulation of Ang II plasma levels

The presence of a local RAS in adipocytes is supported by recent findings showing that RAS is activated during obesity in humans and that obesity-prone rats show increased levels of Ang II and hypertension (84). In mice over-expressing angiotensinogen in adipocytes, the plasma levels of Ang II are increased as well as the systolic blood pressure (90). On the other hand, adipocyte-specific deficiency of angiotensinogen prevented the obesity-induced increase in plasma levels of Ang II (84) indicating an important role of adipocytes on the regulation of Ang II plasma levels and on ulterior consequences including hypertension and vascular remodeling.

### Intracrine action of angiotensin II in the heart and mesenteric arteries

The concept of an intracrine renin–angiotensin aldosterone system (RAAS) in the heart has been substantially supported (3, 5–7, 37). When eplerenone was administered chronically to the failing heart, the intracellular action of Ang II on the inward calcium current (91) was abolished, an effect reversed by aldosterone and related to a decrease of intracellular AT1 receptor levels (91). The activation of the intracrine RAAS might be involved in the generation cellular hypertrophy (92, 93), cardiac arrhythmias (60), and on regulation of vascular tone (94).

Of particular interest was the recent finding that intracellular administration of Ang II to arterial myocytes isolated from mesenteric arteries of Sprague Dawley rats increased the total potassium current and the resting potential, whereas extracellular administration of Ang II reduced total potassium current and elicited depolarization of smooth muscle cells (94). These effects of intracellular Ang II on potassium current and membrane potential were inhibited by dialyzing a PKA inhibitor inside the cell together with Ang II (94). Because it is well known that the resting potential is a determinant factor on the regulation of vascular tone (95), these results might indicate that endogenous or internalized intracellular Ang II in vascular resistance vessels counteracts the effect of extracellular Ang II and plays an important role on the regulation of vascular tone and peripheral resistance (94).

### Mitochondria and intracrine renin-angiotensin system

A revealing finding was that in the ER, renin cleaves angiotensinogen to Ang I, which is subsequently processed to Ang II by ACE (96). Different components of the RAS including the processing enzymes, angiotensins, and their receptors can be transported intracellularly via secretory vesicles to the cell surface, to mitochondria, or to the nucleus.

Activation of the mitochondrial Ang system is coupled to mitochondrial nitric oxide (NO) production and the binding of Ang II to mtAT2Rs stimulates NO formation through mtNOS, suppressing mitochondrial oxygen consumption. Nuclear Ang II can stimulate NO formation (via AT2Rs) or Ca^2+^ and phosphoinositol 3 kinase (PI3K) via AT1Rs (96). The pathophysiological meaning of the presence of renin or Ang II in mitochondria is not known, but considering the role of Ang II on oxidative stress, it is possible to think that activation of AT1 or AT2 receptors in mitochondria might be involved in the etiology of heart or kidney failure.

### Cell volume changes everything. Mechanical sensitivity of heart muscle and cardiac remodeling

One of the important limitations on studies of cellular functions is the assumption that cell volume is constant. It is known that preservation of cell volume is fundamental for cell function and survival and that several mechanisms are working constantly in order to maintain cell volume. Changes in metabolism and the transport of osmotically active substances across the cell membrane are important causes of cell volume variations. It is well known that metabolic pathways are sensitive to changes in cell volume and that glycolysis is inhibited by cell swelling [see Ref. (97)]. Cell swelling, which activates several ionic channels at the cell membrane, changes the action potential duration and alters cardiac excitability.

Recently, it has been shown that that the RAAS is involved in the regulation of cell volume in normal as well as in the failing heart (98). Indeed, in myocytes isolated from the failing ventricle and exposed to renin plus angiotensinogen or to Ang II, an increase of cell volume was seen concurrently with the inhibition of the sodium pump (98). The activation of the Na–K–2Cl cotransporter is involved in the effect of Ang II because bumetanide abolished the swelling induced by the peptide (98). Ang II also increases the swelling-dependent chloride current (I_Clswell_) in the failing and in the normal heart (98), while Ang (1–7), which has been found to counteract many effects of Ang II (99), reduces the heart cell volume and decreases the swelling-activate chloride current (I_Clswell_) (98). This effect of the heptapeptide might be involved in the beneficial effect of Ang (1–7) by decreasing the incidence of cardiac arrhythmias during ischemia/reperfusion (65, 100, 101).

Experimental studies using low doses of aliskiren in hypertensive TGR(mRen2) 27 rats, revealed a decreased structural and electrical cardiac remodeling independently of blood pressure (102) supporting the notion that the renin inhibitor has a direct effect on the heart. The beneficial effect of aliskiren was related to a decrease of AT1 receptor levels. Because AT1 receptors are mechanosensors (103) independently of Ang II [see also Ref. (104)], it is reasonable to think that mechanical stress is able to produce cardiac remodeling even in absence of the peptide. These findings leads to the hypothesis that cardiac remodeling elicited by pressure overload, depends upon the mechanical sensitivity of the cardiac muscle to mechanical stimulation (102) determined by the expression of mechanosensors like AT1 receptors.

### On the role of ACE2

Angiotensin-converting enzyme 2 is a newly discovered enzyme having a high homology to ACE and able to hydrolyze Ang II to the peptide Ang (1–7) (105, 106). Ang (1–7) counteracts the pressor effects of Ang II as well as the proliferative and profibrotic effects of the peptide (65, 69, 70, 99, 100, 107, 108), reduces the incidence of heart failure after myocardial infarction in rats (99) and humans (109), and enhances the cardiac function, coronary perfusion, and aortic endothelial function (99). Previous studies have shown that Ang (1–7) increases the conduction velocity in the failing heart (100, 101) and decreases the incidence of slow conduction and reentry. Recently, evidence has been provided that the activation of the ACE2-Ang (1–7)-Mas receptor axis is involved in the regulation of heart cell volume (110) as well as in the magnitude of the swelling-activated chloride current I_(Clswell)_. This effect of Ang (1–7) was inhibited by ouabain, supporting the view that the heptapeptide activates the sodium pump. Ang II, on the other hand, had an opposite effect on heart cell volume causing cell swelling and increasing the swelling-activated chloride current (110). During myocardial ischemia, cell swelling elicited by the inward movement of water increases I_(Clswell)_ with consequent decrease of cardiac refractoriness. These observations support the notion that the activation of the ACE2-Ang (1–7)-Mas receptor axis is of benefit reducing the cell volume and the incidence of cardiac arrhythmias during ischemia-reperfusion (110). In other studies, it was found that the loss of ACE2 accelerates the maladaptive LV remodeling after myocardial infarction (111). Interestingly, perinuclear immunostaining of the Ang (1–7) was found in mesangial cells (112) and very low concentrations of Ang (1–7) stimulated NO release opening the possibility that intracellular Ang (1–7) has also an intracrine effect.

Although compelling evidence has been presented supporting the view that ACE2 activation counteracts the effects of Ang II in ventricular muscle, some fundamental aspects of the biological significance of ACE2-Ang (1–7)-Mas receptor axis remain unclear. Overexpression of ACE2 in the failing heart, for instance, does not prevent the progression of human heart failure (109). In human coronary circulation, the levels of Ang (1–7) were found to be linked to those of Ang I not Ang II, indicating no role of ACE2 on Ang II metabolism (113). This finding is not in agreement with previous studies on human heart failure showing that ACE2 plays an important role on Ang II metabolism (109).

In the kidney, evidence has been presented that Ang (1–7) causes vasodilation in renal tubuli and counteracts the effect of AT1 receptor activation in several renal diseases such as tubulointerstitial fibrosis, diabetic nephropathy and glomerulonephritis. Under some experimental conditions, however, Ang (1–7) may be harmful by exacerbating renal injury [see Ref. (114)]. This suggests that the state of activation of local RAS, the involvement of non-Mas receptor mediated pathways, or even the dose might explain the discrepant results (65).

## On the Role of Aldosterone and Mineralocorticoid Receptors

There is experimental and clinical evidence that aldosterone causes fibrosis in the cardiovascular system. The RALES trial, for instance, indicated a beneficial effect of spironolactone on morbidity and mortality in patients with heart failure mainly related to the decrease of fibrosis (115) RALES. The contribution of aldosterone to the effect of local RAS activation has been supported by several studies [see Ref. (3)] and justifies the concept of a local RAAS. Although the expression of aldosterone synthase as well as the synthesis of aldosterone seems unlikely in the normal heart, it has been reported an enhanced synthesis of aldosterone in the failing heart (116, 117). Furthermore, elevated plasma aldosterone levels are associated with increased incidence of heart attack and stroke (118).

In vascular smooth muscle as well as in immune cells, the local RAAS plays an important role on endothelial dysfunction and contributes to the production of arterial stiffness. In humans with obesity and diabetes, the RAAS is associated with enhanced oxidative stress and inflammation in the vascular tissue supporting the view that the mineralocorticoid receptors play a role on generation of insulin resistance (119). Indeed, basic and clinical studies have demonstrated that elevated plasma aldosterone levels predict the development of insulin resistance by interfering with insulin signaling in vascular tissues. Aldosterone suppresses insulin signaling via the downregulation of insulin receptor substrate-1 in vascular smooth muscle cells (120, 121).

Recent observations indicated that spironolactone enhances the beneficial effect of aliskiren on cardiac structural and electrical remodeling in TGR(mRen2)27 rats (122) and that chronic administration of eplerenone to the failing heart reduces the cardiac effect of Ang II on inward calcium current through a decline in AT1 receptor level at the surface cell membrane (91). Because AT1 receptor is a mechanosensor involved in cardiac remodeling, it is reasonable to think that part of the beneficial effect of spironolactone in the failing heart is related to a smaller sensitivity of cardiac muscle to mechanical stress.

## Summary

Recent findings that Ang II can be hydrolyzed by ACE2 and neprisylin as well the evidence of new receptors for Ang (IV), Ang (1–7), and Ang III, and the possibility that Ang (1–12) might be the mother of all angiotensins are other evidences of how complex is the RAS. The observation that in human coronary circulation, the levels of Ang (1–7) are related to those of Ang I, but not of Ang II, lead to the question whether many aspects of Ang (1–7) pharmacology are different in humans.

The presence of a local RAS in adipocytes and the observation that the RAAS is activated during obesity in humans, seem to demonstrate how important is this local system on the generation of obesity and hypertension.

The relevance of cell volume and mechanical stretch as a regulators of chloride or potassium channels and the role of AT1 receptors as mechanosensors independently of Ang II indicates that during myocardial ischemia or heart failure, abnormalities on the electrical properties of the heart and cardiac remodeling can be produced independently of the RAS but able to alter the effect of Ang II and Ang (1–7). The recent observation that intracellular Ang II counteracts the effects of extracellular Ang II on potassium current and resting potential in mesenteric arteries leads to the question whether internalized or endogenous levels of Ang II in vascular resistance vessels represent an important factor on the regulation of peripheral resistance and arterial blood pressure. Furthermore, evidence that components of the RAS are present in mitochondria and in the nucleus raises the possibility that the activation of AT1 and AT2 receptors in these organelles influences gene expression and oxidative stress, which is an important cause of cellular dysfunction and the cause of several diseases. Further studies on all these areas will provide opportunity to prevent and treat cardiovascular and renal diseases. The possible role of PRRs on the regulation of cellular homeostasis including autophagy as well as the importance of Ang II AT2 receptors on ventricular hypertrophy needs to be clarified.

## Conflict of Interest Statement

The authors declare that the research was conducted in the absence of any commercial or financial relationships that could be construed as a potential conflict of interest.

## Abbreviations

ACE, angiotensin-converting enzyme; ACE2, angiotensin-converting enzyme 2; Ang (1–7), angiotensin (1–7); Ang II, angiotensin II; ARB, angiotensin receptor blocker; ESRD, end-stage renal disease; I_Clswell_, swelling-dependent chloride current; LV, left ventricle; LVH, left ventricular hypertrophy; NO, nitric oxide; RAS, renin–angiotensin system; SHR, spontaneous hypertensive rats.
